# Calculating the Circular Dichroism of Chiral Halide Perovskites: A Tight-Binding Approach

**DOI:** 10.1021/acs.jpclett.3c02705

**Published:** 2023-12-14

**Authors:** Sofia Apergi, Geert Brocks, Shuxia Tao

**Affiliations:** † Materials Simulation and Modelling, Department of Applied Physics, Eindhoven University of Technology, P.O. Box 513, 5600 MB Eindhoven, The Netherlands; ‡ Center for Computational Energy Research, Department of Applied Physics, Eindhoven University of Technology, P.O. Box 513, 5600 MB Eindhoven, The Netherlands; ¶ Computational Chemical Physics, Faculty of Science and Technology, and MESA+ Institute for Nanotechnology, University of Twente, P.O. Box 217, 7500 AE Enschede, The Netherlands

## Abstract

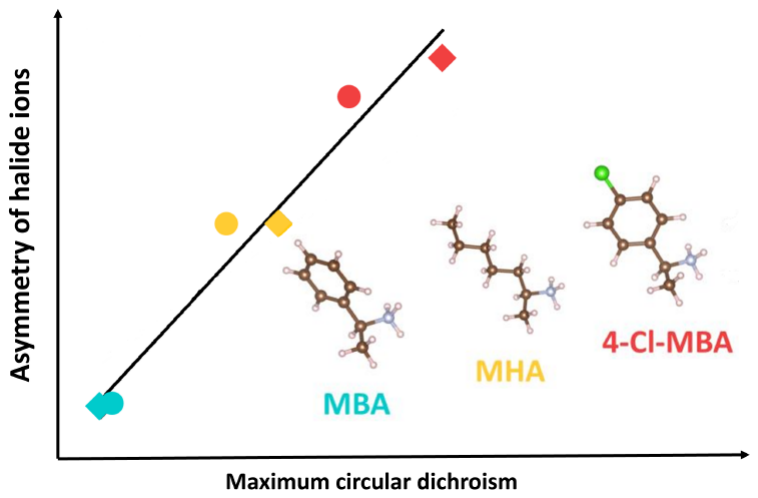

Chiral metal halide perovskites have emerged as promising optoelectronic materials for the emission and detection of circularly polarized visible light. Despite chirality being realized by adding chiral organic cations or ligands, the chiroptical activity originates from the metal halide framework. The mechanism is not well understood, as an overarching modeling framework is lacking. Capturing chirality requires going beyond electric dipole transitions, which is the common approximation in condensed matter calculations. We present a density functional theory (DFT) parametrized tight-binding (TB) model, which allows us to calculate optical properties including circular dichroism (CD) at low computational cost. Comparing Pb-based chiral perovskites with different organic cations and halide anions, we find that the structural helicity within the metal halide layers determines the size of the CD. Our results mark an important step in understanding the complex correlations of structural, electronic, and optical properties of chiral perovskites and provide a useful tool to predict new compounds with desired properties for novel optoelectronic applications.

Circularly polarized light (CPL) is a vital ingredient in many emerging or existing technologies, for instance, in quantum computing,^1,2^ data storage and encryption,^3^ biomolecular sensing,^4,5^ imaging,^6,7^ and asymmetric photochemical synthesis.^8,9^ Chiral metal halide perovskites (called chiral perovskites from here on) have recently garnered extensive attention due to their potential in applications requiring the manipulation of CPL. Chiral perovskites are formed by mixing common perovskite precursors, such as lead or tin halides, with chiral organic cations. This mixing results in the formation of lower dimensional metal halide lattices, often two-dimensional (2D), separated by chiral organic spacers. Chirality transfer arising from interfacing chiral cations with metal-halide layers breaks the mirror symmetry in those layers.^10,11^ The materials then exhibit optical absorption and luminescence that is sensitive to the circular polarization of the light, in other words chiral dichroism (CD), making them ideal candidates for applications that involve CPL emission and detection.^10−17^

With the increasing interest in chiral perovskites, the understanding of the mechanisms that govern their optical properties is lagging somewhat behind. Most of the attention so far has been given to the spin-dependent electronic properties of chiral perovskites, specifically to chirality-induced spin selectivity (CISS).^18^ A theoretical study emphasizes an interplay between spin–orbit coupling (SOC) and chirality to explain CISS and expands this explanation to CD.^19^ It has also been argued that similar phenomena may be found in nonchiral perovskites if their symmetry gives rise to Rashba splitting.^20^ Sidestepping the detailed mechanisms behind CISS or CD, ref (21) demonstrates a correlation between the size of the chirality-induced spin-splitting and the size of the in-plane asymmetric distortion of the metal halide octahedra, using crystallographic and first-principles studies.

The mechanisms behind, specifically, the chiroptical activity in halide perovskites are unclear. Whereas SOC plays a foundational role in explaining CISS, its role in CD is uncertain. Ultimately structure, a lack of symmetry in particular, should govern these effects, but whether the electronic and optical properties are decided by the same structural parameters is open to question. Atomistic simulations are ideal for identifying the structural changes chiral molecules introduce in achiral perovskites. However, it is currently challenging to calculate chiroptical activity in solid state materials due to the absence of an established computational framework,^22^ similar to the frameworks that are available for chiral molecular systems.

Calculations for molecules typically focus on the rotatory strength based on the Rosenberg expression.^23−26^ The latter requires transition matrix elements of electric and magnetic dipole operators and involves an averaging over molecular orientations, under the assumption that each orientation is equally probable. In a crystal, orientations are fixed to the lattice apart from vibrations; therefore, orientational averaging is not appropriate.

In this work, we present a computationally efficient method for studying the optical properties of crystalline materials using a density functional theory (DFT)-parametrized tight-binding (TB) model and apply this method specifically to calculate the CD of 2D chiral perovskites. The TB model allows us to calculate band structures and single particle wave functions, including the effects of SOC, at a low computational cost. From there, optical response functions, such as dielectric functions and absorption coefficients, are calculated. The routinely used electric dipole approximation is not sufficient for calculating the main property of interest here, the CD. The latter requires including higher order terms, i.e., magnetic dipole/electric quadrupole. Note that, because of orientational averaging, in molecular calculations contributions to the rotatory strength of the transition matrix elements of the electric quadrupole operator average out to zero, but when fixing the orientation, as is typical for a crystal, they can become as important as those of the magnetic dipole operator.^26^ We have included both magnetic dipole and electric quadrupole contributions.

Molecular calculations of the rotatory strength are often at the level of time-dependent DFT (TDDFT).^24−26^ Whereas, in principle, TDDFT calculations should also be possible for the crystalline materials discussed here, the complexity of chiral 2D halide perovskite structures would make such calculations computationally quite expensive. In contrast, a TB calculation of the optical response functions is much cheaper. By comparing four chiral perovskites with varying structural distortions, we determine the structural parameters that control the features of the CD and discuss the trends when changing the chiral cations or halide anions.

We start from DFT calculations on archetype chiral 2D perovskites, (R/S–MBA)_2_PbI_4_, with an orthorhombic unit cell consisting of four formula units. The structure is shown in Figure 1(a) as an example. It is fully relaxed using the PBE-D3-BJ exchange-correlation functional; details are given in the Computational Methods section. The band structure calculated without SOC (Figure S1) is then used to construct a tight-binding (TB) model on the basis of Wannier orbitals, using Wannier90 software.^27^ The Wannier orbital basis of our TB model comprises Pb 6s and 6p and I 5p orbitals. These are the orbitals that dominate the upper valence band and the lower conduction band states; see Figure S2. To account for SOC, on-site Hamiltonian matrices are added to the TB Hamiltonian matrix, which act on the Pb 6p and I 5p orbitals, respectively. Their general form is
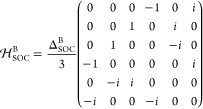
1where  is presented on the basis of {|*p*_*z*_*↑*⟩, |*p*_*z*_*↓*⟩, |*p*_*x*_*↑*⟩, |*p*_*x*_*↓*⟩, |*p*_*y*_*↑*⟩, |*p*_*y*_*↓*⟩}. For B = Pb, the SOC coupling parameter is Δ_SOC_^Pb^ = 1.18 eV, and for B = I, it is Δ_SOC_^I^ = 1.06 eV, as proposed in ref.^28^ In total, our TB model has a basis set consisting of 128 atomic orbitals.

**Figure 1:**
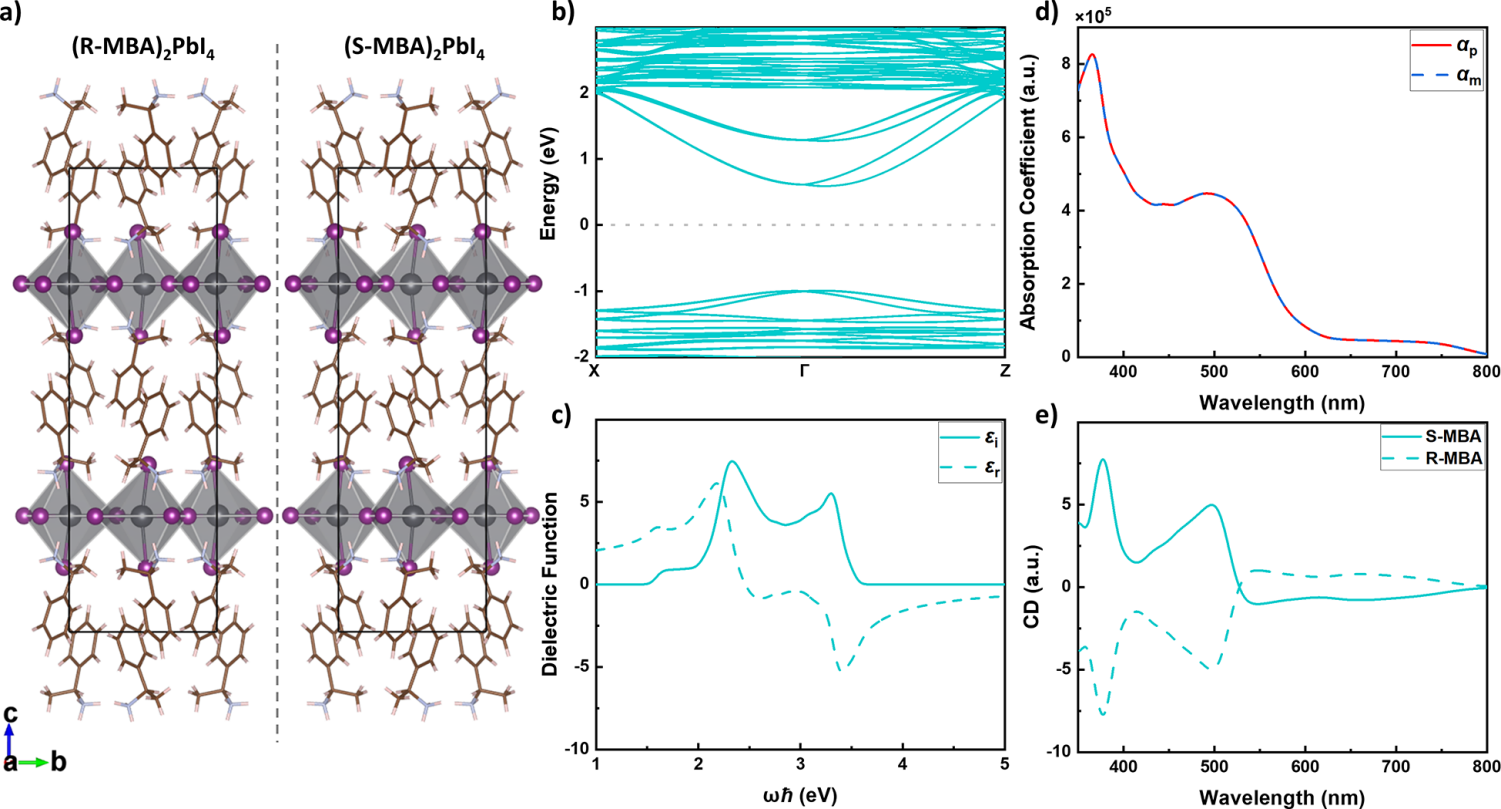
a) Atomistic representation of the R- (left) and S- (right) MBA_2_PbI_4_ perovskites. b) S–MBA_2_PbI_4_ band structure with SOC as calculated from TB. c) Real and imaginary dielectric function, d) absorption coefficient, and e) CD of the (R/S–MBA)_2_PbI_4_ perovskite calculated within the electric quadrupole - magnetic dipole approximation with the inclusion of SOC effects.

The TB band structures are in very good agreement with the ones calculated with DFT; examples are given in Figure S2 (without SOC) and (with SOC) for (S–MBA)_2_PbI_4_. The band gap calculated without SOC (2.16 eV) is very close to the experimental value (2.14 eV),^29^ but inclusion of SOC, in particular on Pb atoms, splits and shifts the lowest conduction band and reduces the band gap considerably,^30^ in our case by 0.56 eV (Figure 1(b)). In principle, the experimental band gap can be recovered using hybrid exchange-correlation functionals or *GW* calculations.^31^ However, the computational costs are high; therefore, we refrain from this step. The effects of varying the band gap will be discussed later. The SOC-induced splitting of the conduction bands near Γ stems from the breaking of the mirror symmetry in chiral perovskites. Its effect on the optical properties is also discussed below.

Having constructed the TB model, we proceeded with the calculation of the optical properties. In this paper, we focus on chiral dichroism (CD), which is basically the difference in the absorption between left- and right-handed CPL. The absorption coefficient is given by the expression
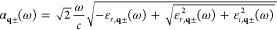
2where ε_*r*_ and ε_*i*_ are the real and imaginary parts of the relative electric permittivity; ω, ***q***, and ± indicate the frequency, the wave vector, and the polarization (right and left circular) of the electromagnetic radiation. We calculate the imaginary part ε_*i*_ using the standard first-order perturbation theory independent-particle expression, neglecting local field effects
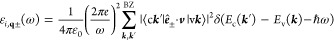
3

The real part ε_*r*_ is then determined using a Kramers-Kroning transform. In eq 3, the indices c and v refer to wave functions of the conduction and valence band, respectively, where ***k***, ***k***′ refer to points in the Brillouin zone (BZ); ***v*** is the velocity operator of an electron (or hole), and  is the polarization vector of the electromagnetic radiation; *E*_c_(***k***′), *E*_v_(***k***) are the electron and hole energies, respectively. The matrix elements  are key quantities from a computational point of view. They are calculated in the approximation
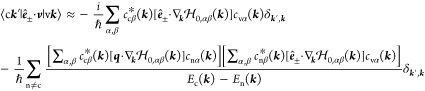
4Here *c*_nα_(***k***) and *E*_n_(***k***) are the TB eigenvectors and eigenvalues respectively, with n the band index and v, c particular values of n;  is the TB Hamiltonian matrix, whose gradient with respect to ***k*** can be determined analytically; indices α and β refer to the orbitals that constitute the basis of the TB model. Sixteen bands are considered in the calculations, 8 valence and 8 conduction bands, and a 50 × 50 × 1 k-point grid is used.

Typical calculations of optical properties use only the first term on the right-hand side of eq 4, which corresponds to the electric dipole (ED) approximation. Electric dipole transitions give by far the dominant contributions to optical absorption, but they are insufficient for calculating CD. The ED approximation assumes a homogeneous electric field across the sample, and such a field does not make a distinction between left and right rotation. Restoring the spatial wave character of the electromagnetic field to first order allows one to make this distinction and can be used to calculate CD.^26^ This corresponds to the electric quadrupole/magnetic dipole (EQMD) approximation, which is represented by the second term on the right-hand side of eq 4.

The derivation of eq 4 can be found in the Supporting Information. Note that we use the velocity form of the transition matrix elements, which has no (gauge) origin dependence when used with Bloch states; see eq 10 in the Supporting Information. We apply the Weyl gauge, where the scalar potential is set to zero, and electric and magnetic fields can be obtained from the vector potential only.

Figure S3 illustrates that while the ED approximation provides a fairly accurate dielectric function and absorption coefficient, the corresponding CD is zero. Including the EQMD term, the dielectric function and absorption spectrum are almost unchanged. That is because the EQMD matrix element is weaker than the ED matrix element by a factor of lattice vector/wavelength ≈10^–3^.^32^ However, whereas the EQMD term is relatively unimportant in calculating the dielectric function and the absorption spectrum, it does supply the CD spectrum. Such a comparison is shown in Figure 3.

The calculated dielectric function of R/S–MBA_2_PbI_4_ is shown in Figure 1(c). Its imaginary part ε_*i*_(ω) is reminiscent of the joint density of states (JDOS), with an onset at *ℏω* = 1.6 eV, and distinct peaks at ∼2.3 and 3.3 eV. The onset corresponds to the band gap as calculated with DFT. Van Hove singularities tend to be prominent in quasi-2D band structures and markedly contribute to peaks in the JDOS.^32^ As R/S–MBA_2_PbI_4_ is indeed a quasi-2D structure, it suggests that the peaks at ∼2.3 and 3.3 eV correspond to Van Hove singularities in the JDOS for transitions from the upper valence bands to the two lowest conduction bands.

The absorption coefficient of R/S–MBA_2_PbI_4_, calculated from eq 2, is shown in Figure 1(d). Qualitatively, the onset and peak structure of the absorption coefficient follow that of ε_*i*_(ω), with an enlarged high frequency part, and the peaks shifted to a slightly higher frequency, because of the influence of ε_*r*_(ω) in eq 2. The calculated CD spectrum, Figure 1(e), shows the typical oscillations and sign changes (the Cotton effect) also observed in the experimentally measured CD spectra. In our case, the extrema correspond to the peaks in the absorption spectrum. Molecular calculations often invoke (interacting) localized excitations to model the Cotton effect.^33^ Here, the excitations are delocalized over the lead halide framework, which prohibits such an interpretation.

Figure 1(e) also demonstrates that the calculated CD signal of S–MBA_2_PbI_4_ is minus that of R–MBA_2_PbI_4_, as it should. As the calculations on the two enantiomers have been totally independent of one another, this serves as a consistency check on the results of the calculations. We have also checked that a calculation with our method on an achiral 2D perovskite, such as BA_2_PbI_4_, gives a zero CD.

While the order of magnitude of the calculated CD is in good agreement with experimental data, a more direct comparison with experiments is unfortunately hard to make. Our model for calculating the CD assumes a single crystal. Experimental CD spectra depend on the thickness of the studied films and are also sensitive to other parameters, such as in-plane homogeneity.^34^ Specifically, CD depends on the relative directions of the electromagnetic wave vector and the crystal axes; so in a multicrystalline film where the grains have a variety of orientations, one observes an averaged signal. In principle, one might assess the optical properties of such films with optical models for light propagation, using permittivities, such as those calculated in the present paper, as input.

As mentioned above, as our calculation starts from a DFT band structure, the band gap is too small, and correspondingly, the onset energy of optical absorption (1.58 eV) is smaller than in experiment (2.14 eV).^29^ We have tested the influence of the band gap on the CD spectra using a scissors operation to increase the TB band gap to the experimental value. Results are shown in Figure S4. Apart from an obvious shift of the absorption onset to a smaller wavelength, the CD spectrum appears slightly more compressed, which is largely the result of the frequency-dependent prefactors in eq 2 and eq 3. Other than these two differences, the CD spectra for different band gaps are very similar. As our main focus in this paper is on the relation between the perovskite structure and CD spectrum, we leave the band gap problem to a future study.

SOC has played a central role in discussing CD in halide perovskites in the literature so far.^19−21^ The results displayed in Figure 1 are indeed calculated by including SOC. To examine how SOC affects the calculated CD, we repeat the calculation, omitting the SOC matrices of eq 1 in the TB Hamiltonian. In this case, only 8 (4 valence and 4 conduction) bands are considered in the calculations, instead of 16 as was the case with SOC. The results of this calculation are shown in Figure 2. As is well-known, SOC splits the bands, where the splitting is particularly large in the lowest conduction band, which has dominant Pb p character, Figure 2(a). Artificially enforcing the same band gap (1.58 eV) on the calculations with and without SOC (again, by means of a scissors operation) facilitates comparison of the optical spectra in Figure S6. In both cases, the structure of ε_*i*_(ω), Figures 1(c) and 2(b), is mainly determined by that in the respective JDOSs, see Figure S5. The peaks observed here carry over to extrema in the CD, Figures 1(e) and 2(c).

**Figure 2:**
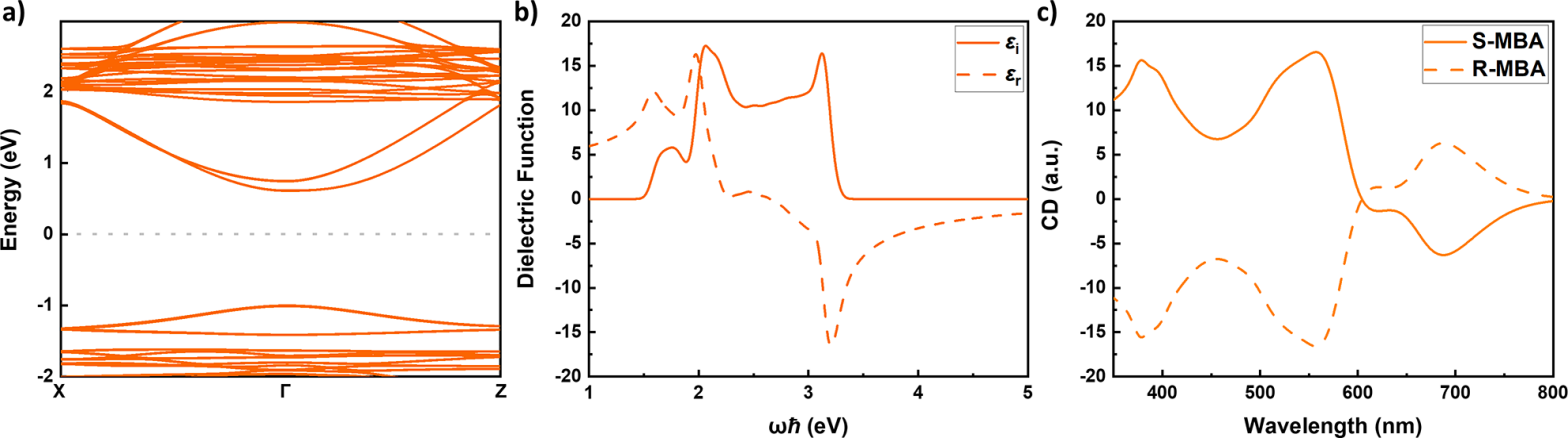
a) S–MBA_2_PbI_4_ band structure without SOC as calculated from TB. b) Real and imaginary dielectric function and c) CD of the (R/S–MBA)_2_PbI_4_ perovskite calculated within the electric quadrupole - magnetic dipole approximation without the inclusion of SOC effects.

Omitting SOC still gives a nonzero CD, Figure 2(c), the size of which is at least of comparable magnitude to the CD calculated with SOC, Figure 1(e). We conclude from this that SOC is not vital to explain the occurrence of CD in halide perovskites, although it is important quantitatively. In fact, for R/S–MBA_2_PbI_4_, the maximum CD calculated without SOC is larger than that calculated with SOC. Apparently, in this case, SOC dilutes the oscillator strengths of the optical transitions somewhat.

Next, we looked at whether there is a relation between the sizes of the CD and the SOC-induced spin splitting in the band structure. The latter determines the CISS effect, so it allows one to assess the correlation between CISS and CD. We calculate the CD of different perovskites with the same space group but with spin splittings of different size. By doing so, differences in crystal symmetry are excluded as the cause of variations in the optical properties. According to ref.,^21^ the chiral perovskites MHA_2_PbI_4_ and 4-Cl–MBA_2_PbBr_4_ belong to the same space group as our benchmark MBA_2_PbI_4_, but MHA_2_PbI_4_ has a much smaller spin-splitting, whereas 4-Cl–MBA_2_PbBr_4_ has a much larger one. Besides having a different A cation, the latter compound also has a different halide anion compared to the benchmark. To separate the effects of the two substitutions, we also construct an artificial 4-Cl–MBA_2_PbI_4_ compound with the same crystal structure as 4-Cl–MBA_2_PbBr_4_ and reoptimize the cell parameters and ionic positions.

The optimized atomic structures of the studied chiral perovskites are shown in Figure S7. Since the three iodine-derived perovskites belong to the same space group and comprise the same inorganic layers, all structural differences stem from the different organic cations (Figure S8). Structural differences resulting from different anions then follow from the substitution of iodine by bromine in 4-Cl–MBA_2_PbX_4_. Structural differences result in different electronic structures (in particular, the band splitting), as well as in a significant variation of the optical properties. The effect of SOC on the band structure of these compounds is typically characterized by Δ*E*_*max*_, which gives the maximum splitting in the lower conduction bands.^21^ It ranges from Δ*E*_*max*_ = 0.04 eV in MHA_2_PbI_4_ to 0.4 eV in 4-Cl–MBA_2_PbI_4_, with both MBA_2_PbI_4_ and 4-Cl–MBA_2_PbBr_4_ having Δ*E*_*max*_ = 0.3 eV, see Figure S8.

Figure 3 gives the CD spectra of the four compounds, MHA_2_PbI_4_, MBA_2_PbI_4_, 4-Cl–MBA_2_PbI_4_, and 4-Cl–MBA_2_PbBr_4_, for light traveling along one of the optical axes. The latter coincide with the crystal axes ***a***, ***b***, and ***c*** in an orthorhombic structure, denoted by Cartesian directions *x*, *y*, and *z* in the following. Note that *x* and *y* are directions in the Pb-halide planes, and *z* is perpendicular to those planes. The maximum spin-splitting discussed above occurs in the lower conduction bands dispersing in the *y*-direction.^21^

**Figure 3:**
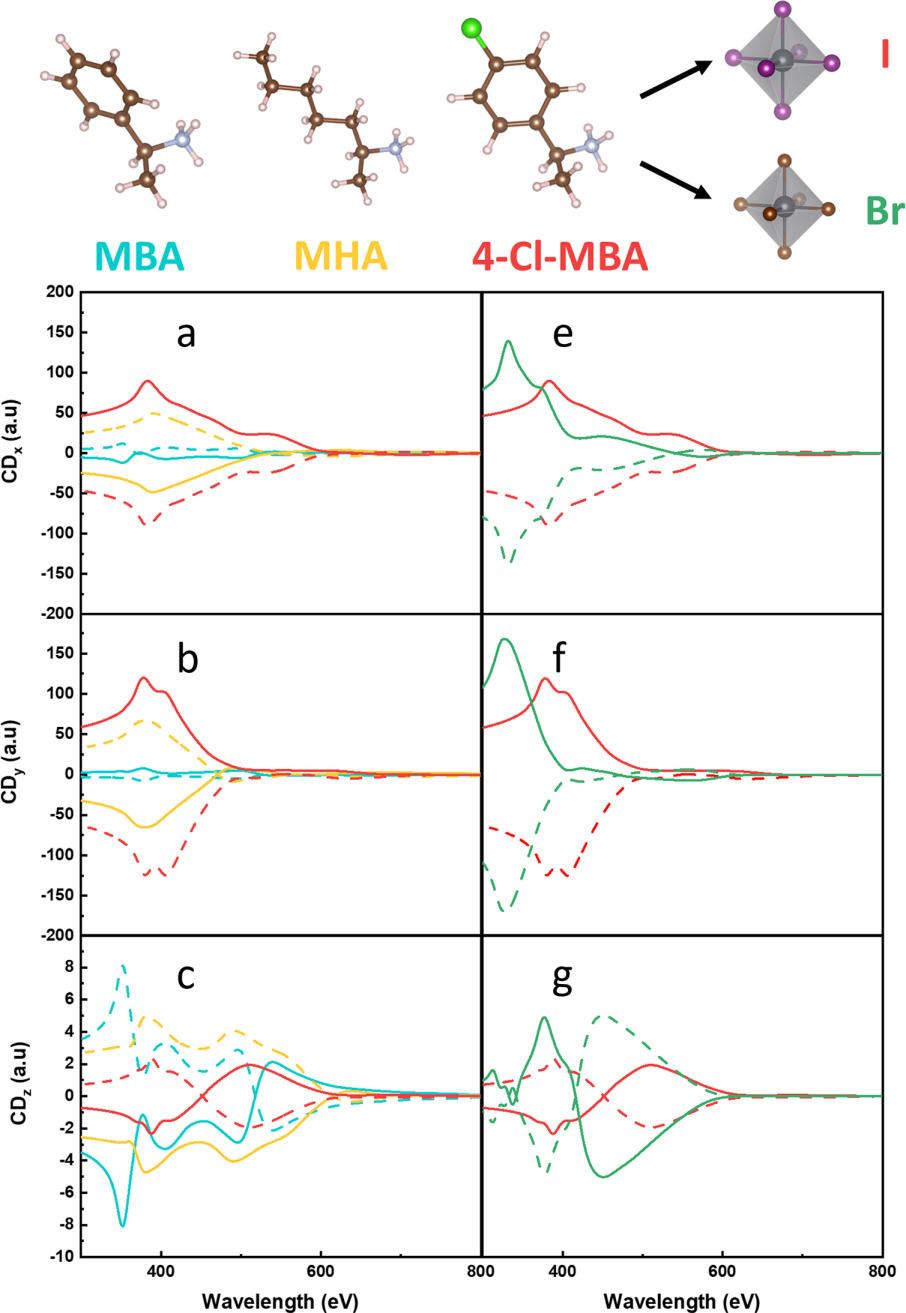
TB-calculated CD spectra, including SOC. The plots are color-coded as follows: yellow for MHA_2_PbI_4_, blue for MBA_2_PbI_4_, red for 4-Cl–MBA_2_PbI_4_, and green for 4-Cl–MBA_2_PbBr_4_. Subfigures a-c compare the three ligands, and subfigures e-g highlight the comparison when the halide ions change from I to Br. Results in the three sets of subfigures a and e, b and f, and c and g distinguish the directions from which light travels, i.e. *x*-, *y*-, and *z*-directions, respectively. The calculated CD along the *z*-direction is much smaller than the *x*- and *y*-directions but is plotted on a different scale for clarity.

According to Figure 3, of the iodide-based compounds, 4-Cl–MBA_2_PbI_4_ has the largest maximum CD in that direction, with that in MHA_2_PbI_4_ being a factor of 2 smaller, and the maximum CD of MBA_2_PbI_4_ more than ten times smaller than that of 4-Cl–MBA_2_PbI_4_. The maximum CD of the bromide-based compound 4-Cl–MBA_2_PbBr_4_ is ∼50% larger. There is no obvious correlation between the size of the CD and that of the spin-splitting discussed above, which is another indication that spin effects are not driving the chiroptical activity.

Figure 3 also shows the calculated CD spectra along the other two principal directions, *x* and *z*. Interestingly, along the *x*-direction, the size of the CD is comparable to that in the *y*-direction, while it also follows the same trend in the order MBA < MHA < 4-Cl–MBA. In fact, the CD spectra of all compounds along the *x* and *y* directions are fairly similar. The main difference is that for all compounds, the maximum CD is slightly lower along the *x* than along the *y*-direction, apart from the CD of MBA_2_PbI_4_, which is higher along *x*.

Comparing different compounds, one observes that apart from the amplitude the CD spectra in the *x*- and *y*-directions of the iodide-based ones are quite similar. In comparison, the spectrum of the bromide-based compound is shifted to a smaller wavelength, as a result of the increased band gap of this compound. The CD spectra in the *z*-direction (perpendicular to the Pb halide planes) are markedly different from those in the in-plane directions. For MHA_2_PbI_4_, 4-Cl–MBA_2_PbI_4_, and 4-Cl–MBA_2_PbBr_4_, the amplitude of the CD in the *z*-direction tends to be at least an order of magnitude smaller. Only for MBA_2_PbI_4_ is the CD comparable in size to that in the *x*- and *y*-directions, but for this compound, the CD in all directions is small anyway, as compared to the other compounds.

One would like to find a correlation between the structure of the compound and the amplitude of the CD. Inspired by ref.,^21^ seven different structural parameters, based on Pb halide bond angles and distances, are calculated, and the results are listed in Table S1. In agreement with ref.,^21^ we find that the spin splitting correlates with the parameter Δβ, which is the difference between two adjacent in-plane Pb–X–Pb bond angles β′ and β″ (Figure S9). However, none of these structural parameters follow the same trend as that of the calculated CD.

This implies that other features of the structures should be connected to the CD. Zooming in on the structure of a single layer of Pb halide octahedra, Figure 4, one observes that the equatorial halide ions do not exactly lie in one plane nor do the axial halide ions. Based on this observation, we define three simple structural parameters, Δ_z–Pb–eq_, Δ_z–X–eq_, and Δ_z–X–ax_, which correspond to the amplitude of the displacements along the *z*-direction of the equatorial Pb, equatorial halide, and axial halide atoms, respectively (Figure 4). As can be seen in Table 1, while Δ_z–Pb–eq_ does not really correlate with the CD, the other two structural parameters follow the same trend as the maximum CD along *x* and *y*, for the three iodide-based perovskites. This means that they increase in the order of MBA < MHA < 4-Cl–MBA.

**Figure 4:**
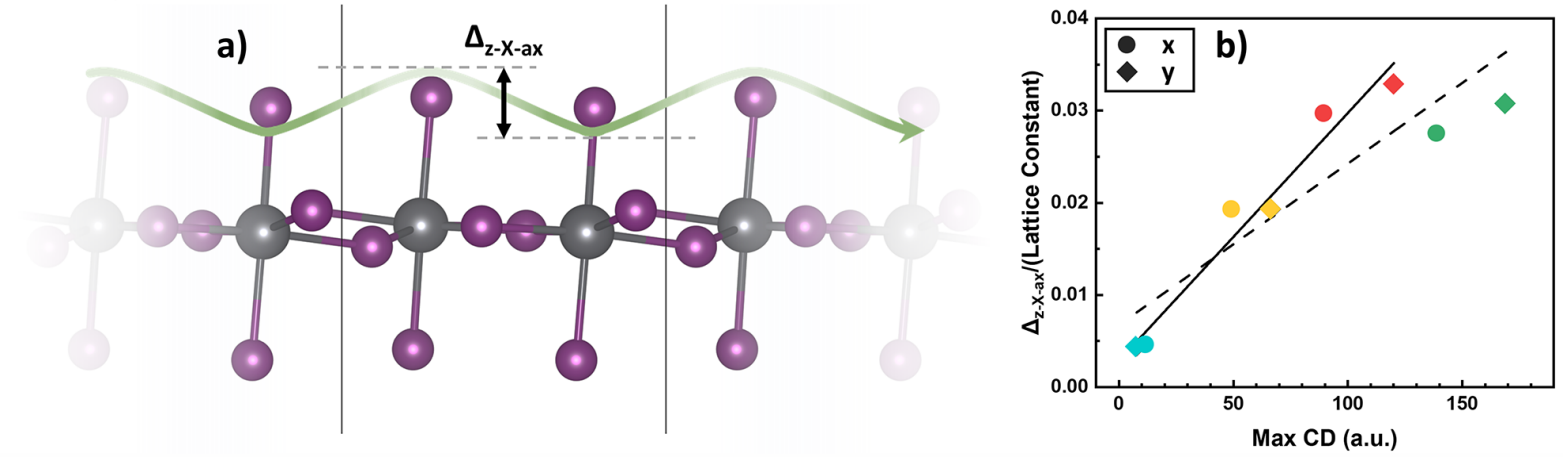
Structural descriptor for characterizing the strength of the CD: A schematic description of the structural parameter Δ_z–X–ax_ (a) and the correlation of the parameter Δ_z–X–ax_ divided by the lattice parameter vs maximum CD (b). The solid and dashed lines correspond to linear fitting without and with taking the CD of the Br-based perovskite into account. The color-coding scheme in (b) corresponds to the one used for the four chiral perovskites in Figure 3.

**Table 1:** CD Maxima along the Three Principal Directions, Structural Parameters Δ_z–Pb–eq_, Δ_z–X–eq_, Δ_z–X–ax_, and Δ*r*_HB_, and Lattice Parameters in Å for the Chiral Perovskites MHA_2_PbI_4_, MBA_2_PbI_4_, 4-Cl–MBA_2_PbI_4_, and 4-Cl–MBA_2_PbBr_4_

Spacer	CD_x,max_	CD_y,max_	CD_z,max_	Δ_z–Pb–eq_	Δ_z–X–eq_	Δ_z–X–ax_	Δ*r*_HB_	***a***	***b***	***c***
MBA	11.93	7.74	8.10	0.05	0.03	0.04	0.017	8.74	9.12	28.60
MHA	49.58	66.71	4.94	0.17	0.09	0.17	0.030	8.81	8.80	33.72
4-Cl-MBA (I)	89.90	120.09	2.33	0.17	0.52	0.27	0.064	9.09	8.22	34.95
4-Cl-MBA (Br)	139.43	169.10	5.02	0.12	0.52	0.24	0.060	8.70	7.79	35.40

According to the same table, smaller lattice parameters also tend to give rise to a larger CD. Interestingly, we can unify the CD amplitudes observed in the *x*- and *y*-directions. If we divide the structural parameter Δ_z–X–ax_ by the lattice parameters ***a***and ***b*** and plot it against the maximum CD along the *x*- and *y*- directions respectively, we get a trend that is very close to linear, see Figure 4. This construction does not work for the other two parameters, Δ_z–Pb–eq_ and Δ_z–X–eq_, see Figure S10. We also observed a correlation between the distances separating the hydrogen atoms from NH_3_^+^ and the halide ions and the calculated CD signal strengths. Specifically, shorter distances are associated with larger CD signals, a relationship previously established in a recent study by Son et al.^35^ This suggests that this parameter reflects the same chiral distortion phenomenon discussed in our analysis above.

The maximum CD along the *z*-direction also tends to increase with a decreasing lattice parameter. Apart from this simple observation, we have not been able to identify a structural parameter that correlates exactly with this trend. However, the CD amplitude in the *z*-direction is relatively small, compared to those in the *x*- and *y*-directions.

Including 4-Cl–MBA_2_PbBr_4_ one observes that, while the calculated CD obeys the same trend as the iodide-based compounds, described by Δ_z–X–ax_/lattice parameter. However, the relation is not exactly linear anymore (Figure 4). This can be partly explained by the fact that the increase in the band gap shifts CD peaks to smaller wavelengths and increases the size of the peaks slightly, a similar effect as is observed in Figure S6. This demonstrates that the features of the CD can be tuned not only by changing the chiral ligands but also by modifying the inorganic layers.

To summarize, in this work, we present a method for calculating the optical properties of chiral halide perovskites. To ensure computational efficiency, our method is based on a TB model, parametrized from DFT calculations and includes on-site SOC Hamiltonians. While similar approaches are used for the calculation of optical properties, of various materials, most studies use the electric dipole approximation, which is not suitable for calculating chiral properties, such as CD. For this reason, we determine higher order terms, namely, the electric quadrupole and magnetic dipole terms, which allow us to calculate the CDs of the chiral perovskites.

We perform calculations on a number of chiral perovskites, varying either chiral ligands or halide anions, and analyze and compare their structural, electronic, and optical properties. In contrast to frequent assumptions, we find no correlation between CD and the chirality-induced spin splitting, suggesting that the two are controlled by different structural parameters. While in-plane inorganic metal-halide octahedral tilting distortions generally determine the size of the spin splitting, out-of-plane ionic displacements of the metal-halide layer are the decisive factor for the size of CD. Specifically, the latter correlates with the out-of-plane displacement amplitude of the axial halide atoms divided by the lattice parameter in the direction of the incident light. The chemical composition of the inorganic layer is another important factor in determining the CD. Replacing I with Br within the same structure increases the size of the CD, in addition to shifting the peaks in the CD spectrum.

Our method is computationally efficient and flexible, making it a suitable starting point for refinements to include effects that have not yet been considered, such as excitonic effects obtained from solving the Bethe-Salpeter equation, or the coupling with lattice dynamics to take the impact of finite temperatures into account. The availability of such a tool therefore creates opportunities to understand more complex interactions of light, spin, and charge in this fascinating class of materials and to design new compositions with new functionalities.

## Computational Methods

Density functional theory calculations were performed using the Projector Augmented Wave (PAW) method, as implemented in the Vienna Ab-Initio Simulation Package (VASP).^36−39^ The electronic exchange-correlation interaction was described by the functional of Perdew, Burke, and Ernzerhof (PBE) within the generalized gradient approximation (GGA)^40^ and energy and force convergence criteria of 10^–5^ eV and 0.02 eV/Å, respectively, were used in all calculations. The D3 correction with Becke-Jonson damping was employed to account for the van der Waals interactions due to the presence of the organic species.^41^ The calculations were performed with a 4 × 4 × 1 Γ-centered k-point grid and a kinetic energy cutoff of 500 eV. Initial structures were acquired from ref.^21^ and subsequently optimized. During geometry optimization, unit cells of four formula units were allowed to fully relax. In all cases, the cell angles remained at 90°, except for MBA_2_PbI_4_, where after full relaxation, the cell angles slightly deviated from 90°. In the latter case, the orthorhombic structure was reinforced, and the positions of the ions were allowed to relax.

## Supporting Information Available

Chiral dichroism calculation, Figures S1–S10, and Table S1 (PDF)
