# Supplementary material for: Calculating the Circular Dichroism of Chiral Halide Perovskites: A Tight-Binding Approach
Source: J Phys Chem Lett. 2023 Dec 14;14(51):11565–72. doi: 10.1021/acs.jpclett.3c02705 (PMC10758117; doi:10.1021/acs.jpclett.3c02705)
Supplement: Supplementary file 1 — jz3c02705_si_001.pdf [file jz3c02705_si_001.pdf]

Calculating the Chiral Dichroism of Halide Perovskites: A Tight-Binding Approach: Supplementary Information

Sofia Apergi,[†,‡] Geert Brocks,[†,‡,¶] and Shuxia Tao[*,†,‡]

†*Materials Simulation and Modelling, Department of Applied Physics, Eindhoven University of Technology, P.O. Box 513, 5600 MB Eindhoven, The Netherlands*

‡*Center for Computational Energy Research, Department of Applied Physics, Eindhoven University of Technology, P.O. Box 513, 5600 MB Eindhoven, The Netherlands*

¶*Computational Materials Science, Faculty of Science and Technology and MESA+, Institute for Nanotechnology, University of Twente, P.O. Box 217, 7500 AE Enschede, The Netherlands*

E-mail: S.X.Tao@Tue.nl

Chiral Dichroism Calculation

To derive the equations that will allow us to calculate the desired properties, i.e. the optical absorption and eventually the chiral dichroism (CD), we start from a one-electron Hamiltonian in absence of any external field

$$\mathcal{H}_0 = \frac{|\boldsymbol{p}|^2}{2m} + V(\boldsymbol{r}) + \xi(\boldsymbol{r})(\boldsymbol{S} \times \boldsymbol{r}) \cdot \boldsymbol{p}, \tag{1}$$

with the last term on the right-hand side describing the spin-orbit coupling. It is obtained from the more familiar $\boldsymbol{L} \cdot \boldsymbol{S}$ expression by writing $\boldsymbol{L} \cdot \boldsymbol{S} = (\boldsymbol{r} \times \boldsymbol{p}) \cdot \boldsymbol{S} = (\boldsymbol{S} \times \boldsymbol{r}) \cdot \boldsymbol{p}$. The

Hamiltonian that describes the motion of an electron in an external electromagnetic field with vector potential $\boldsymbol{A}$ according to "minimal coupling" is then[1]

$$\mathcal{H} = \frac{[\boldsymbol{p} + e\boldsymbol{A}]^2}{2m} + V(\boldsymbol{r}) + \xi(\boldsymbol{r})(\boldsymbol{S} \times \boldsymbol{r}) \cdot (\boldsymbol{p} + e\boldsymbol{A}) \approx \mathcal{H}_0 + \mathcal{H}_{\text{e−R}}, \tag{2}$$

with $\mathcal{H}_0$ denoting the unperturbed one-electron Hamiltonian, Eq. 1, and $\mathcal{H}_{\text{e−R}}$ describing the interaction of an electron with the electromagnetic radiation

$$\mathcal{H}_{\text{e−R}} = \frac{e}{2}(\boldsymbol{A} \cdot \boldsymbol{v} + \boldsymbol{v} \cdot \boldsymbol{A}). \tag{3}$$

We have neglected the quadratic term $e^2|\boldsymbol{A}|^2/2m$ in Eq. 2, and have defined the velocity operator

$$\boldsymbol{v} = \frac{\boldsymbol{p}}{m} + \xi(\boldsymbol{r})(\boldsymbol{S} \times \boldsymbol{r}). \tag{4}$$

The electron-radiation interaction in 3 can be treated in first order perturbation. To calculate the dielectric function, we can take advantage of Fermi's golden rule, to calculate the transition probability for an electron from a valence band $|\mathrm{v}\boldsymbol{k}_\mathrm{v}\rangle$ with energy and wave vector E_v and $\boldsymbol{k}_\mathrm{v}$ respectively, to a conduction band $|\mathrm{c}\boldsymbol{k}_\mathrm{c}\rangle$, with energy and wave vector E_c and $\boldsymbol{k}_\mathrm{c}$. If we assume that the vector potential can be described by a monochromatic wave with frequency ω, then the expression for the imaginary part of the (relative) dielectric function becomes[1]

$$\varepsilon_i(\omega) = \frac{1}{4\pi\varepsilon_0}\left(\frac{2\pi}{\omega}\right)^2 \sum_{\boldsymbol{k}_\mathrm{c},\boldsymbol{k}_\mathrm{v}} |\langle \mathrm{c}\boldsymbol{k}_\mathrm{c}|\mathcal{H}_{\text{e−R}}|\mathrm{v}\boldsymbol{k}_\mathrm{v}\rangle|^2 \delta(E_\mathrm{c}(\boldsymbol{k}_\mathrm{c}) - E_\mathrm{v}(\boldsymbol{k}_\mathrm{v}) - \hbar\omega) \tag{5}$$

After determining $\varepsilon_i(\omega)$, the real part of the dielectric function, $\varepsilon_r(\omega)$, can be calculated using the Kramers-Kroning relations. Finally, the absorption coefficient is given by the expression

$$\alpha(\omega) = \sqrt{2}\frac{\omega}{c}\sqrt{-\varepsilon_r(\omega) + \sqrt{\varepsilon_r^2(\omega) + \varepsilon_i^2(\omega)}} \tag{6}$$

The CD can then be evaluated by considering the difference in absorption between left- and right-handed polarized light.

Key quantities from a computational point of view are the oscillator strengths in Eq. 5, $|\langle \mathrm{c}|\mathcal{H}_{\mathrm{e-R}}|\mathrm{v}\rangle|^2$. We assume a monochromatic plane wave with vector potential

$$\boldsymbol{A}(\boldsymbol{r},t) = \hat{\boldsymbol{e}}\; A_0 \exp\left[i(\boldsymbol{q}\cdot\boldsymbol{r}-\omega t)\right] - i\hat{\boldsymbol{e}}^*\; A_0^* \exp\left[-i(\boldsymbol{q}\cdot\boldsymbol{r}-\omega t)\right] \tag{7}$$

where $\hat{\boldsymbol{e}}$ is the polarization vector, A_0 is the amplitude, $\boldsymbol{q}$ the wave vector, and ω the frequency. As an example, if $\boldsymbol{q} = q\hat{\boldsymbol{z}}$, then $\hat{\boldsymbol{e}} = \hat{\boldsymbol{x}} \pm i\hat{\boldsymbol{y}}$ represents left and right circular polarized light. Writing the Bloch functions for the electrons in the conduction and valence bands, respectively, as

$$|\mathrm{c}\boldsymbol{k}_{\mathrm{c}}\rangle = u_{\mathrm{c},\boldsymbol{k}_{\mathrm{c}}}(\boldsymbol{r})\exp\left[i(\boldsymbol{k}_{\mathrm{c}}\cdot\boldsymbol{r})\right] \tag{8}$$

and

$$|\mathrm{v}\boldsymbol{k}_{\mathrm{v}}\rangle = u_{\mathrm{v},\boldsymbol{k}_{\mathrm{v}}}(\boldsymbol{r})\exp\left[i(\boldsymbol{k}_{\mathrm{v}}\cdot\boldsymbol{r})\right], \tag{9}$$

with $u_{\mathrm{v,c},\boldsymbol{k}_{\mathrm{v,c}}}$ cell-periodic functions, it can be shown that the relevant matrix element can be written as

$$\langle \mathrm{c}\boldsymbol{k}'|\mathcal{H}_{\mathrm{e-R}}|\mathrm{v}\boldsymbol{k}\rangle = \delta_{\boldsymbol{k}',\boldsymbol{k}+\boldsymbol{q}}\frac{eA_0}{\Omega}\int_{\Omega} u^*_{\mathrm{c},\boldsymbol{k}+\boldsymbol{q}}(\boldsymbol{r})\left[\hat{\boldsymbol{e}}\cdot\boldsymbol{v}\right]u_{\mathrm{v},\boldsymbol{k}}(\boldsymbol{r})d\boldsymbol{r}, \tag{10}$$

where $\delta_{\boldsymbol{k}',\boldsymbol{k}+\boldsymbol{q}}$ ensures the conservation of momentum in the absorption process. The integration is over the unit cell, with volume Ω. Assuming that the size of the wave vector $|\boldsymbol{q}|$ is much smaller than the scale set for $|\boldsymbol{k}|$, which is the Brillouin zone, then $u_{\mathrm{c},\boldsymbol{k}+\boldsymbol{q}}$ can be expanded into a Taylor series in $\boldsymbol{q}$

$$u_{\mathrm{c},\boldsymbol{k}+\boldsymbol{q}}(\boldsymbol{r}) \simeq u_{\mathrm{c},\boldsymbol{k}}(\boldsymbol{r}) + \boldsymbol{q}\cdot\nabla_{\boldsymbol{k}}u_{\mathrm{c},\boldsymbol{k}}(\boldsymbol{r}) + ... \tag{11}$$

We expect this to be accurate if the radiation is in the visible (or lower frequency) region.

Electric Dipole Approximation

Very often in calculations of optical properties, only the first term of the expansion in 11 is considered, which leads to the so-called electric dipole (ED) approximation

$$\langle \mathrm{c}\boldsymbol{k}|\mathcal{H}_{\mathrm{e-R}}|\mathrm{v}\boldsymbol{k}\rangle = \frac{eA_0}{\Omega}\int\limits_{\Omega} u^*_{\mathrm{c},\boldsymbol{k}}(\boldsymbol{r})\,[\hat{\boldsymbol{e}}\cdot\boldsymbol{v}]\,u_{\mathrm{v},\boldsymbol{k}}(\boldsymbol{r})d\boldsymbol{r} \tag{12}$$

To simplify calculating this matrix element, one can make use of the relation

$$\boldsymbol{v} = \frac{1}{i\hbar}\left[\boldsymbol{r},\mathcal{H}_0\right], \tag{13}$$

which is easily derived from Eqs. 1 and 4. Expressing the Bloch states (8,9) on a tight-binding basis, we have

$$u_{n,\boldsymbol{k}}(\boldsymbol{r}) = e^{-i\boldsymbol{k}\cdot\boldsymbol{r}}\sum_{\alpha}\mathrm{c}_{n\alpha}(\boldsymbol{k})\chi_{\alpha\boldsymbol{k}}(\boldsymbol{r}); \quad \chi_{\alpha\boldsymbol{k}}(\boldsymbol{r}) = \frac{1}{\sqrt{N}}\sum_{\boldsymbol{R}} e^{i\boldsymbol{k}\cdot(\boldsymbol{R}+\boldsymbol{t}_\alpha)}\phi_\alpha(\boldsymbol{r}-\boldsymbol{R}-\boldsymbol{t}_\alpha). \tag{14}$$

Here $\chi_{\alpha\boldsymbol{k}}(\boldsymbol{r})$ is a Bloch orbital, and $\phi_\alpha(\boldsymbol{r}-\boldsymbol{R}-\boldsymbol{t}_\alpha)$ is an atomic orbital, characterized by the label α, centered at an atomic position $\boldsymbol{t}_\alpha$ within a unit cell at position $\boldsymbol{R}$ in the lattice, and N is the number of lattice sites. Using 13 and 14 in 12 gives, after some algebra[2]

$$\begin{aligned}\langle \mathrm{c}\boldsymbol{k}|\mathcal{H}_{\mathrm{e-R}}|\mathrm{v}\boldsymbol{k}\rangle = {}& i\frac{eA_0}{\hbar}\left[E_{\mathrm{c}}(\boldsymbol{k})-E_{\mathrm{v}}(\boldsymbol{k})\right]\sum_{\alpha,\beta}c^*_{\mathrm{c}\beta}(\boldsymbol{k})\left[\hat{\boldsymbol{e}}\cdot\boldsymbol{r}_{\beta\alpha}\right]c_{\mathrm{v}\alpha}(\boldsymbol{k}) \\ & - i\frac{eA_0}{\hbar}\sum_{\alpha,\beta}c^*_{\mathrm{c}\beta}(\boldsymbol{k})\left[\hat{\boldsymbol{e}}\cdot\nabla_{\boldsymbol{k}}\mathcal{H}_{0,\alpha\beta}(\boldsymbol{k})\right]c_{\mathrm{v}\alpha}(\boldsymbol{k}).\end{aligned} \tag{15}$$

Here $\boldsymbol{r}_{\beta\alpha} = \langle\phi_\beta|\boldsymbol{r}|\phi_\alpha\rangle$. Typically, this term is negligible, unless the orbitals ϕ_β and ϕ_α are on the same atomic site, and even then, the first term on the right-hand side of Eq. 15 is typically negligible compared to the second term, see Ref.[2] The expression $\mathcal{H}_{0,\alpha\beta}(\boldsymbol{k})$ stands for the matrix elements of the tight-binding matrix. As their analytical expressions as functions of $\boldsymbol{k}$ are known, the $\nabla_{\boldsymbol{k}}\mathcal{H}_{0,\alpha\beta}(\boldsymbol{k})$ operations can be done analytically.

Equation 15 in 5 (with $\delta_{\boldsymbol{k}_c,\boldsymbol{k}_v}$, and $\boldsymbol{k}_c = \boldsymbol{k}$) then gives the electric dipole approximation to the dielectric function on a tight-binding basis. Electric dipole transitions give by far the dominant contributions to optical absorption, but they are insufficient for calculating CD. The problem of the electric dipole approximation is that by only keeping the first term of the expansion in 11, one assumes $\boldsymbol{q} = 0$, which implies a homogeneous field, 7. A homogeneous field cannot make a distinction between left and right rotation, and we need some inhomogeneity, i.e., $\boldsymbol{q} \neq 0$ in 7. This problem is solved by including the second term of the expansion, 11, in the calculations, which leads to the electric quadrupole - magnetic dipole (EQMD) approximation.

Electric Quadrupole - Magnetic Dipole Approximation

Within the EQMD approximation, we consider the expansion of 11 up to and including the second term. Instead of 12, we get the expression

$$\begin{aligned}
\langle \mathrm{c}\boldsymbol{k}+\boldsymbol{q}|\mathcal{H}_{\mathrm{e-R}}|\mathrm{v}\boldsymbol{k}\rangle &= \frac{eA_0}{\Omega}\int\limits_{\Omega} u^*_{\mathrm{c},\boldsymbol{k}+\boldsymbol{q}}(\boldsymbol{r})\,[\hat{\boldsymbol{e}}\cdot\boldsymbol{v}]\,u_{\mathrm{v},\boldsymbol{k}}(\boldsymbol{r})d\boldsymbol{r} \\
&\simeq \frac{eA_0}{\Omega}\int\limits_{\Omega} u^*_{\mathrm{c},\boldsymbol{k}}(\boldsymbol{r})\,[\hat{\boldsymbol{e}}\cdot\boldsymbol{v}]\,u_{\mathrm{v},\boldsymbol{k}}(\boldsymbol{r})d\boldsymbol{r} \qquad (16) \\
&+ \frac{eA_0}{\Omega}\int\limits_{\Omega} \boldsymbol{q}\cdot\nabla_{\boldsymbol{k}}u^*_{\mathrm{c},\boldsymbol{k}}(\boldsymbol{r})\,[\hat{\boldsymbol{e}}\cdot\boldsymbol{v}]\,u_{\mathrm{v},\boldsymbol{k}}(\boldsymbol{r})d\boldsymbol{r} \qquad (17)
\end{aligned}$$

The integral of 16 is the ED approximation, evaluated in the previous section, which means only 17 remains to be determined. We can define a Hamiltonian $\mathcal{H}_0(\boldsymbol{k})$ by replacing $\boldsymbol{p}$ in 1 by $\boldsymbol{p}+\hbar\boldsymbol{k}$. The cell-periodic parts of the Bloch functions are eigenfunctions of this Hamiltonian

$$\mathcal{H}_0(\boldsymbol{k})u_{n,\boldsymbol{k}}(\boldsymbol{r}) = E_n(\boldsymbol{k})u_{n,\boldsymbol{k}}(\boldsymbol{r}) \qquad (18)$$

From the expression for $\mathcal{H}_0(\boldsymbol{k})$, it can be easily shown that

$$\boldsymbol{v} + \frac{\hbar\boldsymbol{k}}{m} = \frac{1}{\hbar}\left[\nabla_{\boldsymbol{k}}, \mathcal{H}_0(\boldsymbol{k})\right], \tag{19}$$

and therefore

$$\int_\Omega u^*_{n',\boldsymbol{k}}(\boldsymbol{r})\left(\boldsymbol{v} + \frac{\hbar\boldsymbol{k}}{m}\right) u_{n,\boldsymbol{k}}(\boldsymbol{r})d\boldsymbol{r} = \frac{1}{\hbar}\left(E_n(\boldsymbol{k}) - E_{n'}(\boldsymbol{k})\right)\int_\Omega u^*_{n',\boldsymbol{k}}(\boldsymbol{r})\nabla_{\boldsymbol{k}} u_{n,\boldsymbol{k}}(\boldsymbol{r})d\boldsymbol{r}, \tag{20}$$

for $n \neq n'$. Making use of the fact that the functions $u_{n,\boldsymbol{k}}(\boldsymbol{r})$ form a complete orthonormal set, we can derive the expression

$$\begin{aligned}\nabla_{\boldsymbol{k}} u_{n,\boldsymbol{k}} &= \sum_{n'\neq n} u_{n',\boldsymbol{k}}(\boldsymbol{r})\int_\Omega u^*_{n',\boldsymbol{k}}(\boldsymbol{r})\nabla_{\boldsymbol{k}} u_{n,\boldsymbol{k}}(\boldsymbol{r})d\boldsymbol{r} \\ &= \hbar\sum_{n'\neq n} u_{n',\boldsymbol{k}}(\boldsymbol{r})\frac{\int_\Omega u^*_{n',\boldsymbol{k}}(\boldsymbol{r})\left(\boldsymbol{v} + \frac{\hbar\boldsymbol{k}}{m}\right) u_{n,\boldsymbol{k}}(\boldsymbol{r})}{E_n(\boldsymbol{k}) - E_{n'}(\boldsymbol{k})}. \end{aligned}\tag{21}$$

Note that the $\hbar\boldsymbol{k}/m$ term does not actually give any contribution, as the functions $u_{n,\boldsymbol{k}}(\boldsymbol{r})$ are orthogonal.

This result can be used in 17 to give

$$\begin{aligned}&\int_\Omega \boldsymbol{q}\cdot\nabla_{\boldsymbol{k}} u^*_{\mathrm{c},\boldsymbol{k}}(\boldsymbol{r})\left[\hat{\boldsymbol{e}}\cdot\boldsymbol{v}\right] u_{\mathrm{v},\boldsymbol{k}}(\boldsymbol{r})d\boldsymbol{r} \\ &\qquad = \hbar\sum_{n'\neq \mathrm{c}} \frac{\left(\int_\Omega u^*_{\mathrm{c},\boldsymbol{k}}(\boldsymbol{r})\left[\boldsymbol{q}\cdot\boldsymbol{v}\right] u_{n',\boldsymbol{k}}(\boldsymbol{r})d\boldsymbol{r}\right)\left(\int_\Omega u^*_{n',\boldsymbol{k}}(\boldsymbol{r})\left[\hat{\boldsymbol{e}}\cdot\boldsymbol{v}\right] u_{\mathrm{v},\boldsymbol{k}}(\boldsymbol{r})d\boldsymbol{r}\right)}{E_\mathrm{c}(\boldsymbol{k}) - E_{n'}(\boldsymbol{k})}. \end{aligned}\tag{22}$$

The integrals in 22 are very similar to 12, and they can be evaluated on a tight-binding basis in the same way as discussed in the previous section. As within the EQMD approximation the radiation field is no longer treated as homogeneous, it can be used to calculate CD.

Figures

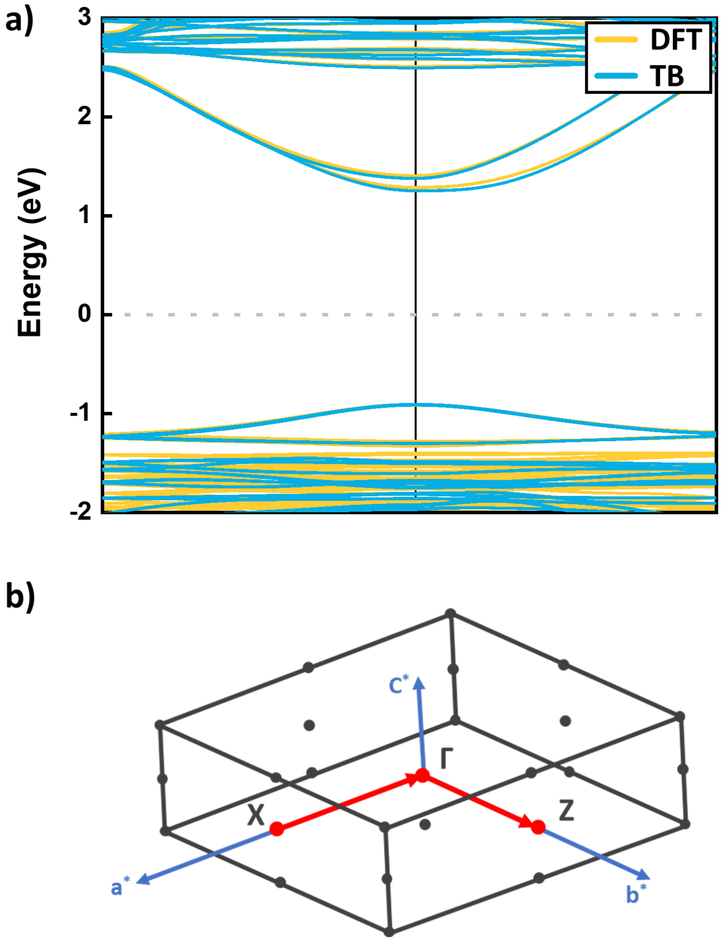

Figure S1: a S−MBA_2PbI_4 band structure calculation without SOC calculated with DFT and with the DFT parametrized TB model. b) High symmetry k-point path used in the band structure calculations.

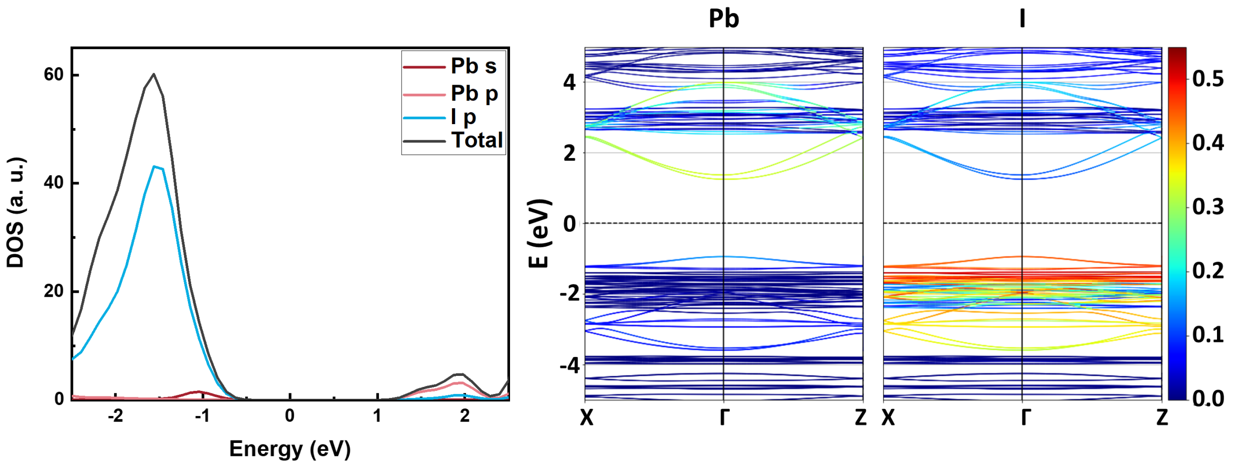

Figure S2: Atomic orbital projected density of states (left) and Pb and I contribution to the electronic band structure (right) from DFT. The electronic bands around the band gap mainly comprise Pb 6s and 6p and I 5p contributions.

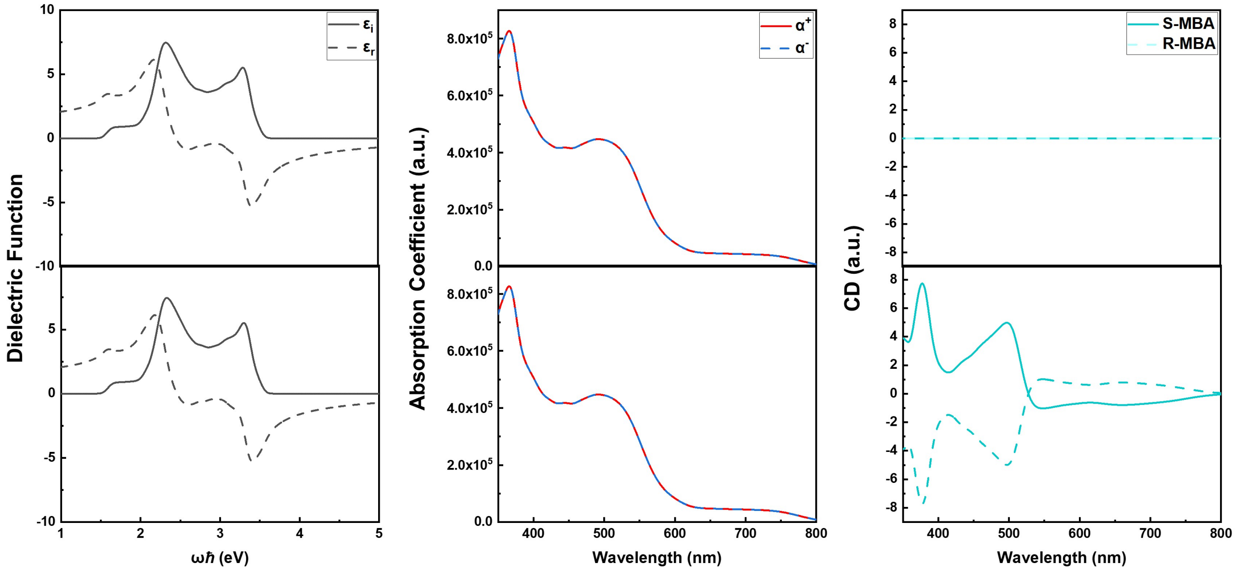

Figure S3: Real and imaginary dielectric function (left), absorption coefficient (middle), and CD ($\alpha^+ - \alpha^-$) (right) of the (R/S−MBA)$_2$PbI$_4$ perovskite calculated within the electric dipole (top) and the electric quadrupole - magnetic dipole (bottom) approximation.

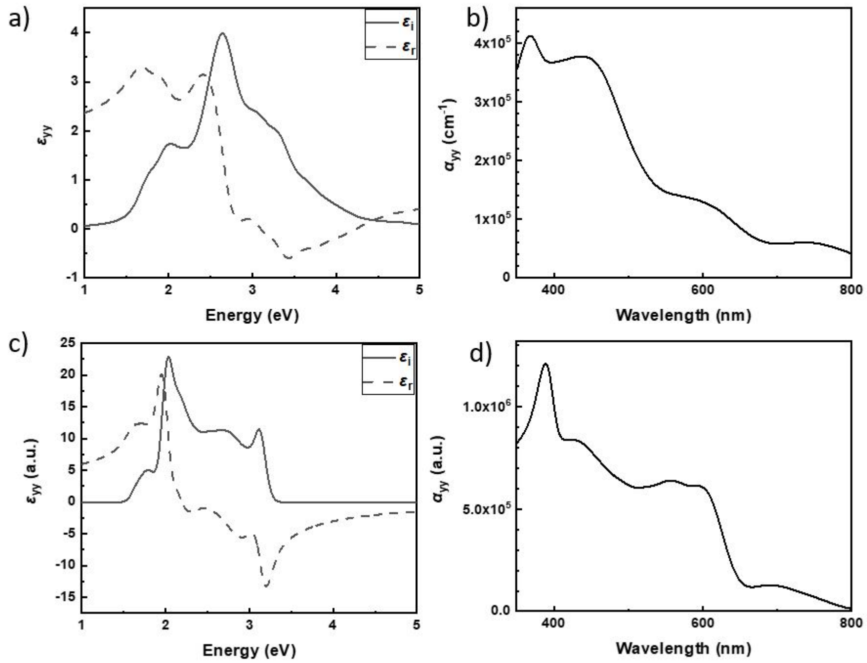

Figure S4: Comparison of DFT calculated dielectric function and absorption coefficient (top panel) with those calculated by TB (bottom panel). Good qualitative agreement can be found at energy range lower than 2 eV and whereas larger deviations is seen in energies higher than 2 eV. This is due to the fact that in the TB model, only 4 bands including 2 conduction bands and 2 valence bands each, were included, whereas in the DFT calculations all bands in the relevant energy range were taken into account. The latter give rise to additional features in the respective spectra.

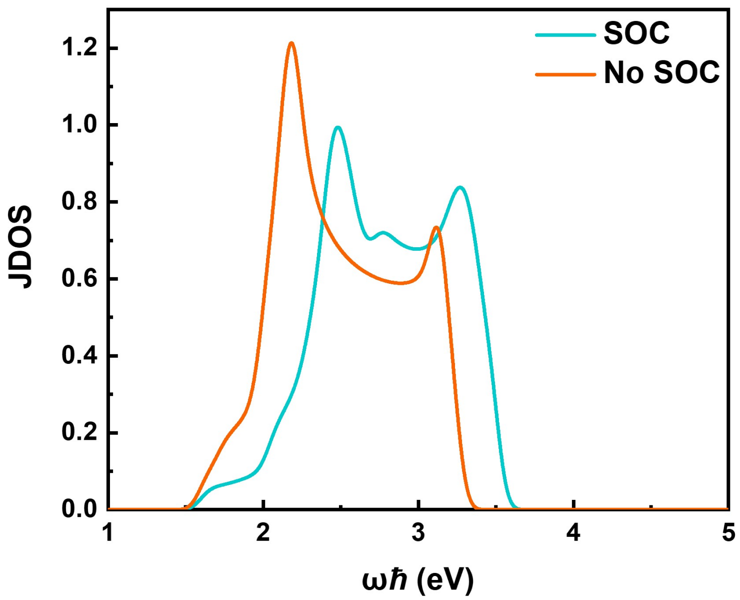

Figure S5: JDOS of the $(R/S-MBA)_2PbI_4$ perovskite calculated within the electric quadrupole - magnetic dipole approximation with and without the inclusion of SOC effects.

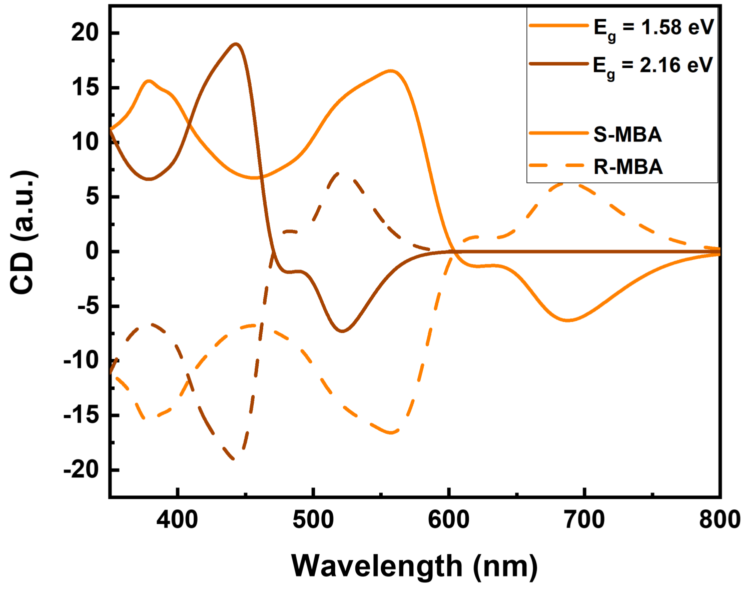

Figure S6: CD of the $(R/S-MBA)_2PbI_4$ perovskite calculated within the electric quadrupole - magnetic dipole approximation without the inclusion of SOC effects, for two different band gap (E_g) values.

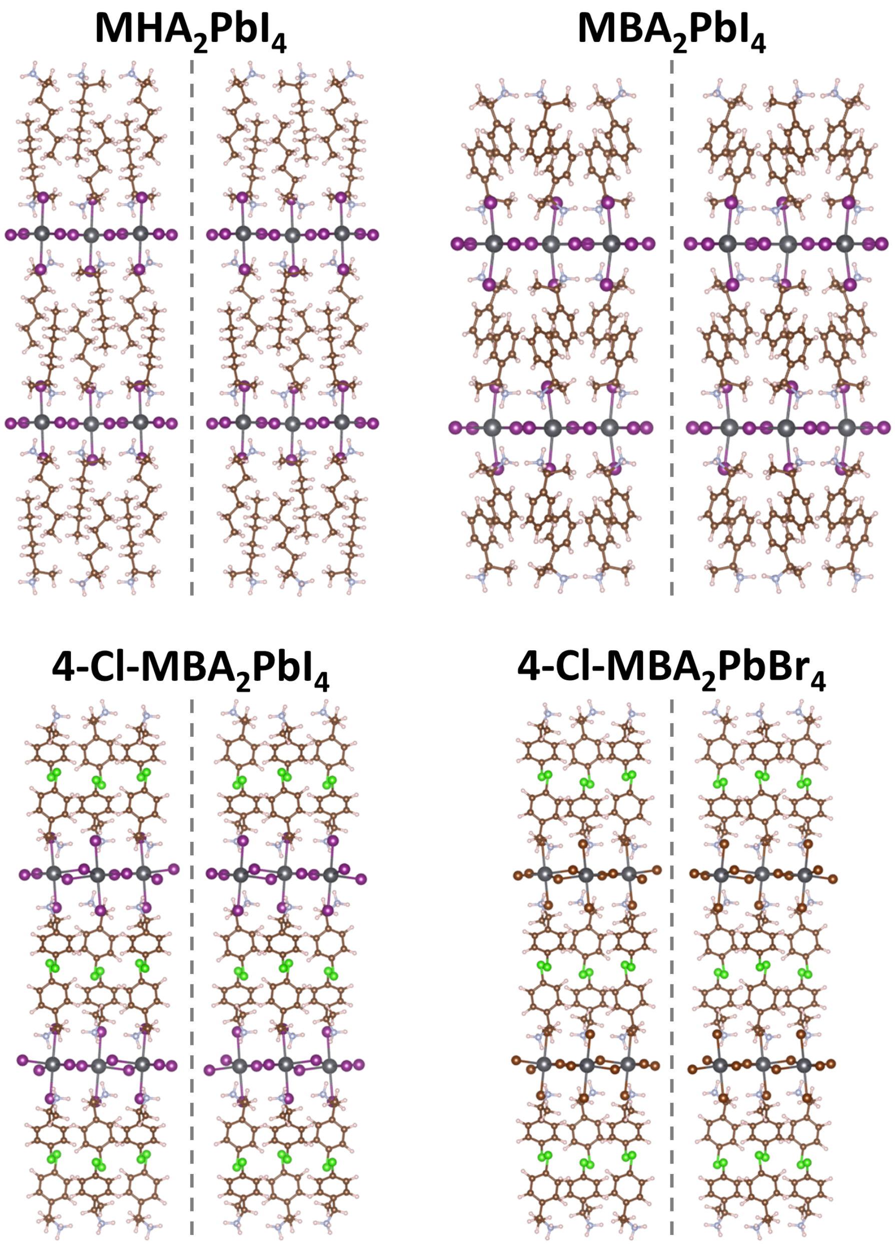

Figure S7: Atomistic representation of the S- (left) and R- (right) enantiomers of the chiral perovskites MHA_2PbI_4, MBA_2PbI_4, $4-Cl-MBA_2PbI_4$, and $4-Cl-MBA_2PbBr_4$.

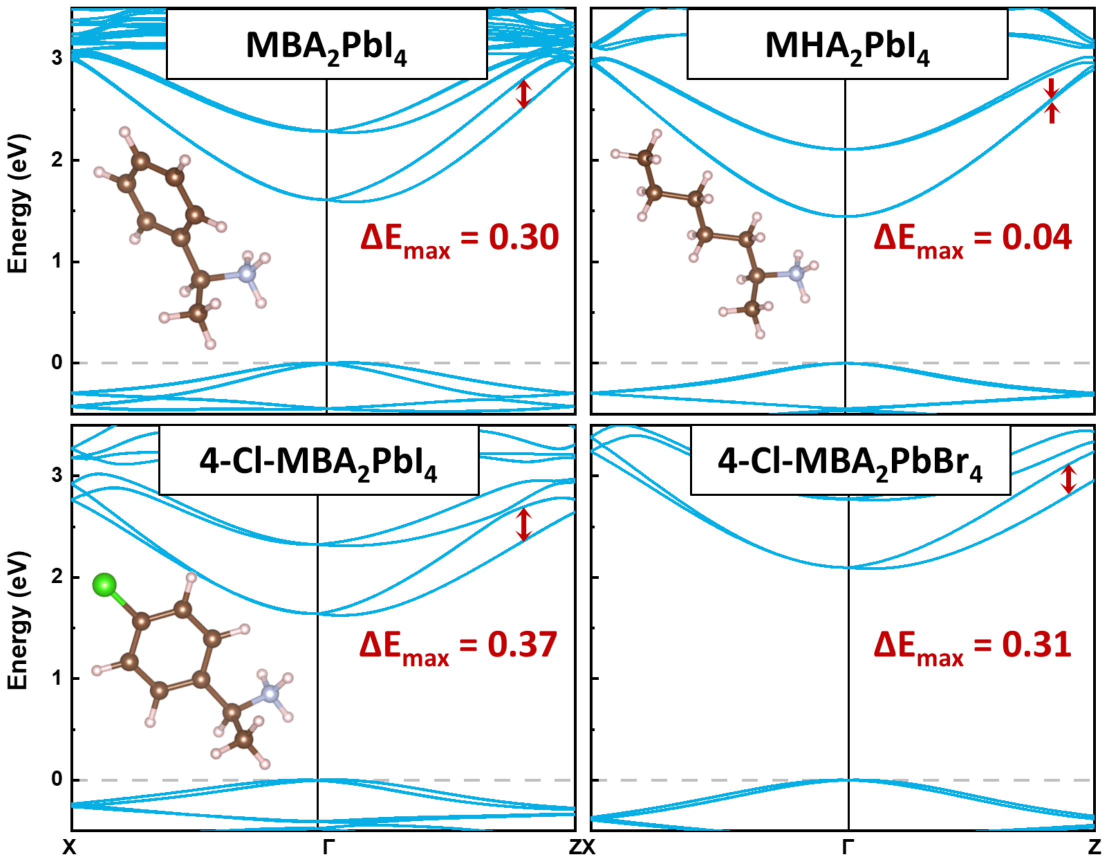

Figure S8: TB-calculated electronic band structures for the chiral perovskites MHA_2PbI_4, MBA_2PbI_4, 4−Cl−MBA_2PbI_4, and 4−Cl−MBA_2PbBr_4 including SOC.

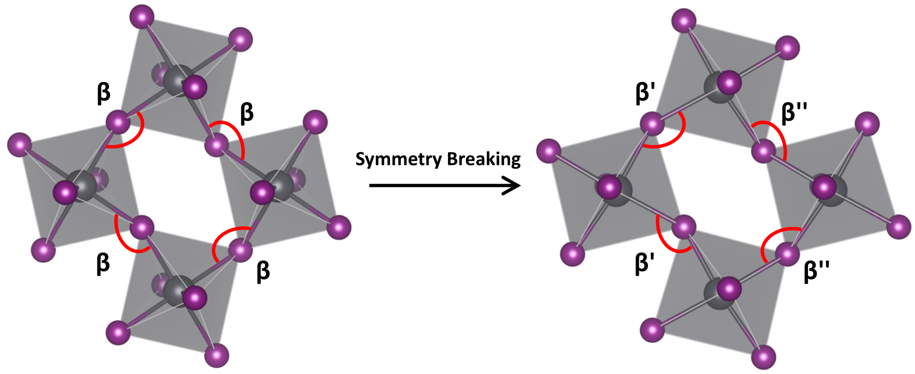

Figure S9: The breaking of mirror symmetry of the PbX_4 layers caused by the chiral organic spacers leads to an asymmetric tilting of the PbX_6 octahedra. With symmetric tilting, all in-plane Pb−X−Pb angles are equal (β), but asymmetric tilting creates two different Pb−X−Pb angles, β' and β''.

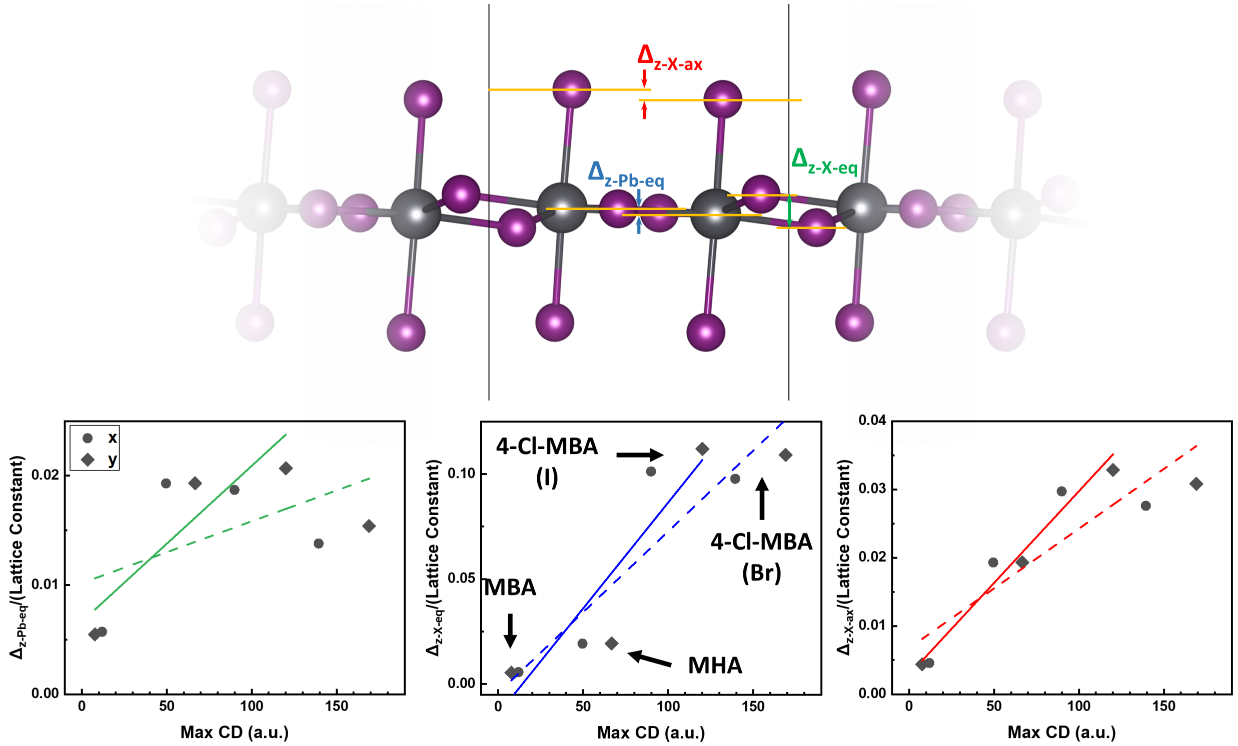

Figure S10: Comparison of the structural parameters $\Delta_{\mathrm{z-Pb-eq}}$, $\Delta_{\mathrm{z-X-eq}}$, and $\Delta_{\mathrm{z-X-ax}}$. The solid and dashed lines correspond to linear fitting without and with taking the CD of the Br-based perovskite into account.

Table

Table S1: Structural parameters and calculated spin-splitting for the chiral perovskites MHA_2PbI_4, MBA_2PbI_4, 4−Cl−MBA_2PbI_4, and 4−Cl−MBA_2PbBr_4. Parameters Δd and σ^2 describe the octahedral distortion and are given by the expressions $\Delta d = (1/6)\sum_i (d_i - d_0)^2/d_i$, where d_i and d_0 are the six different Pb−X bond lengths and their average respectively, and $\sigma^2 = \sum_i (\theta_i - 90)^2/11$, where θ_i are the twelve X−Pb−X angles of an octahedron. $\Delta\beta$ is the difference between adjacent in-plane Pb−X−Pb angles β' and β'' (Figure S8), while $\Delta\beta_{in}$ and $\Delta\beta_{out}$ is the difference of the in- and out-of-plane components of β' and β'', respectively. Finally, D_{in} and D_{out} is the maximum of the difference of β_{in} and β_{out} from 180°. All angles are given in degrees.

**Spacer**	$\boldsymbol{\Delta d}(\times 10^{-5})$	$\boldsymbol{\sigma^2}$	$\boldsymbol{\Delta\beta}$	$\boldsymbol{\Delta\beta_{in}}$	$\boldsymbol{D_{in}}$	$\boldsymbol{\Delta\beta_{out}}$	$\boldsymbol{D_{out}}$	$\boldsymbol{\Delta E_{max}}$
MBA	14.8	22.00	7.67	7.66	33.04	0.25	0.67	0.30
MHA	0.8	12.08	0.63	0.68	29.99	0.39	1.80	0.04
4-Cl-MBA (I)	23.8	48.04	14.39	11.08	38.29	15.07	17.19	0.37
4-Cl-MBA (Br)	40.3	57.8	14.56	11.76	37.56	12.91	16.69	0.31

References

(1) Peter, Y.; Cardona, M. *Fundamentals of Semiconductors: Physics and Materials Properties*; Springer Science & Business Media, 2010.

(2) Pedersen, T. G.; Pedersen, K.; Kriestensen, T. B. Optical Matrix Elements in Tight-Binding Calculations. *Phys. Rev. B* **2001**, *63*, 201101.
